# P-184. Gastrointestinal Illness in Attendees of FIFA Football World Cup 2022 in Qatar

**DOI:** 10.1093/ofid/ofae631.388

**Published:** 2025-01-29

**Authors:** Sherin Shams, Thoraya M Saleh, Hanaa Nafady-Hego, Emad B Ibrahim, Aimon Malik, Anil G Thomas, Samah Saleem, Aftab M Azad, Abdul-Badi Abou-Samra, Adeel A Butt

**Affiliations:** Hamad Medical Corporation, Doha, Ad Dawhah, Qatar; Hamad Medical Corporation, Doha, Ad Dawhah, Qatar; Microbiology and immunology, Faculty of Medicine, Assiut University, Assiut, Egypt., Doha, Ad Dawhah, Qatar; Hamad Medical Corporation, Doha, Ad Dawhah, Qatar; Hamad Medical corporation, Doha, Ad Dawhah, Qatar; Hamad Medical corporation, Doha, Ad Dawhah, Qatar; Hamad Medical corporation, Doha, Ad Dawhah, Qatar; Department of Emergency Medicine, Hamad Medical Corporation, Doha, Qatar, Doha, Ad Dawhah, Qatar; Hamad Medical corporation, Doha, Ad Dawhah, Qatar; Weill Cornell Medicine, Doha, Ad Dawhah, Qatar

## Abstract

**Background:**

Mass gathering events may facilitate transmission of foodborne diseases. We determined the pattern and causative organisms of gastrointestinal illness in the attendees of the FIFA Football World Cup 2022 (FIFA-WC2022), one of the world’s largest sporting events, which was hosted by Qatar.

Table 1. Baseline characteristics of individuals who underwent stool testing.

**Figure:**
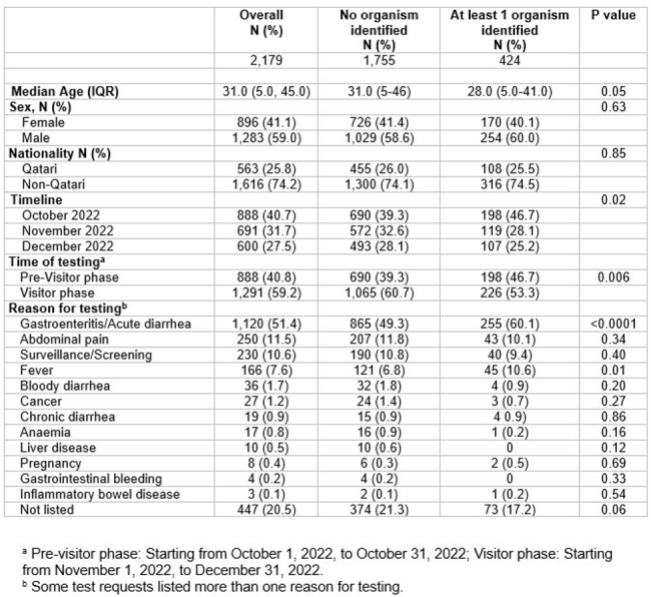

**Methods:**

The study was conducted at Hamad Medical Corporation in Qatar, which was the designated provider and overseer of healthcare during FIFA-WC2022 for all visitors and served as the national reference laboratory for all microbiology testing. We retrospectively retrieved all stool testing data from attendees of FIFA-WC2022 from October 1 to December 31, 2022. Routine stool microscopy for ova and parasite and stool culture and sensitivity tests were performed on all samples received. PCR testing was performed for suspected food poisoning or when a rapid result was required for public health response.

Table 2. List of organisms detected by culture and/or PCR. (Note that the number of organisms is higher than that of the patients as some patients had more than one organism identified). At least one organism was detected in 424 individuals.

**Figure:**
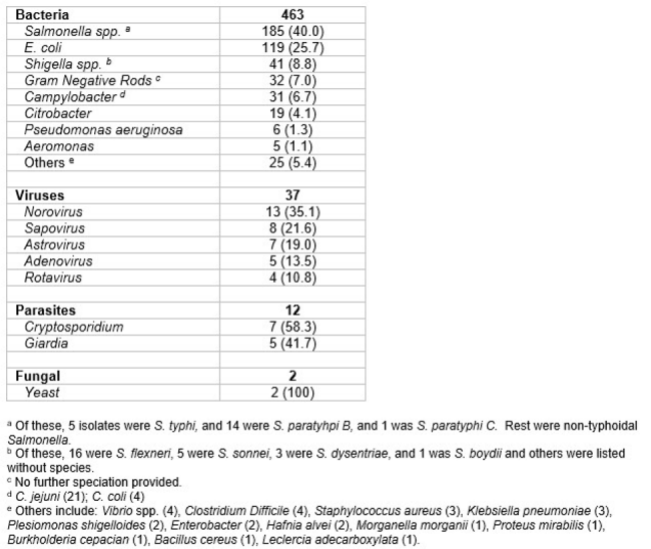

**Results:**

During the FIFA-WC2022, approximately 1.4 million visitors entered Qatar. During the study period, a total of 2,179 stool samples were received for testing. The most common reasons for testing were acute diarrhea/gastroenteritis (51.4%), abdominal pain (11.5%), screening/surveillance of potential contacts (10.6%), and fever (7.6%). At least one organism was identified in 424 cases. The most commonly identified organisms were bacteria (92.5%), viruses (7.8%), and parasites (2.8%). The most frequent bacteria detected were Salmonella spp. (40%), E. coli (25.7%), and Shigella spp. (8.8%).

**Conclusion:**

A relatively small number of individuals attending the FIFA-WC2022 experienced laboratory confirmed infectious gastroenteritis. Bacteria were more frequently isolated than viruses. The yield of stool testing was higher for those presenting with acute diarrhea/gastroenteritis and fever, but not for those with abdominal pain or for surveillance/screening. These results have implications and lessons for planning future mass gathering events.

**Disclosures:**

**Adeel A. Butt, MD, MS**, Gilead Sciences: Grant/Research Support

